# 3D printable gelatin/nisin biomaterial inks for antimicrobial tissue engineering applications†

**DOI:** 10.1039/d4ma00544a

**Published:** 2024-09-10

**Authors:** Mateo Dallos Ortega, Jenny Aveyard, Alexander Ciupa, Robert J. Poole, David Whetnall, Julia G. Behnsen, Raechelle A. D’Sa

**Affiliations:** a School of Engineering, University of Liverpool, Harrison Hughes Building, Brownlow Hill Liverpool L69 3GH UK r.dsa@liverpool.ac.uk; b Materials Innovation Factory, University of Liverpool 51 Oxford Street Liverpool L7 3NY UK

## Abstract

Modern regenerative medicine approaches can rely on the fabrication of personalised medical devices and implants; however, many of these can fail due to infections, requiring antibiotics and revision surgeries. Given the rise in multidrug resistant bacteria, developing implants with antimicrobial activity without the use of traditional antibiotics is crucial for successful implant integration and improving patient outcomes. 3D printed gelatin-based implants have a broad range of applications in regenerative medicine due to their biocompatibility, ease of modification and degradability. In this paper, we report on the development of gelatin biomaterial inks loaded with the antimicrobial peptide, nisin, for extrusion-based 3D printing to produce scaffolds with controlled porosity, high shape fidelity, and structural stability. Rheological properties were comprehensively studied to develop inks that had shear thinning behaviour and viscoelastic properties to ensure optimal printability and extrudability, and enable precise deposition and structural integrity during 3D printing. The 3D printed scaffolds fabricated from the gelatin/nisin inks demonstrated excellent antimicrobial efficacy (complete kill) against Gram positive bacteria methicillin-resistant *Staphylococcus aureus* (*MRSA*). Overall, this ink's high printability and antimicrobial efficacy with the model antimicrobial peptide, nisin, offers the potential to develop customisable regenerative medicine implants that can effectively combat infection without contributing to the development of multidrug resistant bacteria.

## Abstract 2

Antimicrobial peptide loaded biomaterial inks for 3D printing can fabricate personalised medical devices that has significant healthcare benefits given the lower risk infection related failure and lower tendency to develop antimicrobial resistant bacteria.

## Graphical abstract

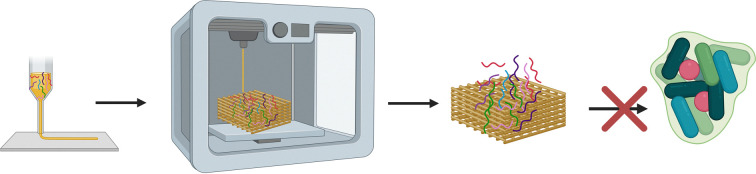

## 1. Introduction

The goal of modern regenerative medicine is to develop implants and devices that can replace damaged or diseased tissue. Infections associated with implanted regenerative medical devices can cause failure of the implant, trauma to the patient and substantial economic burden on healthcare systems.^1^ Infections are generally caused by bacterial pathogens such as *Staphylococcus aureus* and *Pseudomonas aeruginosa* which form biofilms on implant surfaces and can cause failure of the implant, leading to revision surgeries.^1^ Biofilms are intrinsically tolerant to antibiotics such that implant-associated infections are extremely challenging to treat, especially given the rise in antimicrobial resistance (AMR). Antimicrobial resistance (AMR) poses a critical threat to global public health, as evidenced by a recent Lancet report that estimated approximately 1.27 million deaths in 2019 were directly attributed to AMR bacteria.^1^ Moreover, a recent WHO report has projected that by 2050 potentially 350 million deaths will result from AMR-based infections if left unaddressed.^2,3^ This escalating crisis of AMR necessitates the development of alternative antimicrobial treatment modalities. Antimicrobial peptides (AMPs) are part of the innate immune response of most humans, plants and animals and have emerged as a promising alternative antimicrobial agent.^4^ Their advantages over conventional antibiotics stem from their broad-spectrum antimicrobial and antibiofilm activity, low tendency to cause bacterial resistance and ability to modulate the host immune response.^5,6^

AMPs are low molecular-weight cationic oligopeptides with 10–60 amino acids and possess a wide range of inhibitory effects against bacteria and other microorganisms.^4,7–9^ Their activity is based on disrupting bacterial cell membranes, controlling the immune response, and modulating inflammation. AMPs are characteristically rich in cationic (*e.g.* Arg and Lys) and hydrophobic amino acids.^4^ These two components allow AMPs to fold into amphipathic secondary structures and interact with bacterial cell membranes which typically have anionic surfaces rich in lipids.^7^

Whilst AMPs have significant promise as alternatives to antibiotics, they can be cytotoxic if used on their own and can denature under physiological conditions. Therefore, methods to sustainably deliver AMPs to the site of an infection – through hydrogels, nanoparticles and hydrogels – are actively being pursued. Indeed, many hydrogels loaded with antimicrobial agents have been investigated for their feasibility in treating soft tissue infections. Though hydrogels can release antimicrobials over an extended period of time, lack of mechanical strength deters their extensive use, especially in treatments involving reconstructive surgeries. The ability to manufacture customisable antimicrobial hydrogel implants with eluting antimicrobials and mechanical stability would offer a significant advantage over the current state of the art. 3D printing has emerged as a new customisable manufacturing technology in regenerative medicine and tissue engineering applications.^10–12^ Currently, extensive research efforts are being focused on the development of advanced 3D printing methodologies and inks to produce a wide range of materials and shapes.^13^ These advanced 3D printing methodologies encompass laser-based, inkjet-based, and extrusion-based printing. Extrusion-based 3D printing is promising as a wide array of biomaterial inks can be developed to generate tissue substitutes for clinical use.^12^ For example, these 3D printed hydrogels can produce complex hierarchical porosities in personalized shapes that are custom-manufactured through 3D scanning of a defect or wound site.^12^

Many synthetic and naturally occurring hydrogels loaded with antibiotics have been studied for their applicability in treating soft tissue infections and although they release antibiotics over an extended period of time, the lack of mechanical strength deters their extensive use in regenerative medicine applications. Gelatin based hydrogels are used extensively due to their inherent biocompatibility, ease of modification, degradability, and rapid gelation induced by low temperature.^13^ However, optimizing their printability poses several challenges. One key issue is temperature control. At room temperature, gelatin inks solidify rapidly, causing nozzle clogging. Conversely, high temperatures can weaken the gel structure, compromising the integrity and shape fidelity of the printed scaffolds. Achieving high resolution with gelatin inks can also be challenging. The water content in gelatin causes the ink to spread, blurring fine details and limiting the creation of intricate structures. Lastly, the duration of printing sessions presents a challenge. Longer sessions increase the risk of premature gelation within the nozzle, and therefore fast printing techniques and optimized ink formulations are required to ensure the desired results. To this end, we have focused on the development of antimicrobial 3D printable gelatin inks for the fabrication of customisable infection resistant implants without the use of antibiotics.^13^ This represents a significant unmet need, as despite the potential of 3D printing, infection still remains a major hurdle in terms of failure.^13^ Few reports in the literature involving antimicrobial gelatin inks include a study by Liu *et al.*, who developed polyacrylamide/gelatin/silver nanoparticle (PAAm-GelatinAgNPs) inks to improve gelatin-based hydrogels.^13^ The ink is based on double networks, with physically cross-linked gelatin as the first network and covalently cross-linked PAAm as the second network.^13^ Cheng *et al.*, reported the development of a bio adhesive gelatin methacryloyl (GelMA) based ink for 3D printed hydrogels used for infectious wound healing.^14^ Brites *et al.*, developed gelatin-based hydrogel inks with antibacterial manuka honey for wound healing applications.^15^

The aim of this study was to develop 3D printed biomaterial inks for the potential regeneration and infection control of soft tissues. The ideal parameters this ink should possess include appropriate rheological properties to allow ease of printability, shape fidelity after printing, mechanical stability, biological properties (including degradation or insolubility in physiological solutions), cytocompatibility, and non-immunogenicity.^16^ Nisin was used as the model AMP in this study as it is an FDA approved AMP used widely for preservation of food and beverages and it has been shown to be effective in soft tissue infections such as mastitis,^17^ dermatitis^18^ and periodontitis.^19^ The 3D printed hydrogel scaffolds were freeze dried post printing to investigate the feasibility of increasing their shelf-life. The resultant scaffolds were evaluated for the release of nisin by high performance liquid chromatography (HPLC). The antimicrobial efficacy of the scaffolds was tested to determine their ability to target two clinically relevant bacteria, methicillin-resistant *Staphylococcus aureus* (*MRSA*) and *Pseudomonas aeruginosa.* The antimicrobial efficacy testing was carried out over a 4 week period to investigate long-term stability.

## 2. Materials and methods

### 2.1. Materials

Gelatin (type A) from bovine skin with 175 g bloom, nisin from *Lactococcus lactis*, glutaraldehyde 50 wt% in H_2_O solution, ethanol, nutrient agar (NA), nutrient broth (NB), Luria broth (LB), and Luria agar (LA) were purchased from Sigma-Aldrich.

### 2.2. Gelatin–nisin ink preparation

**Fig. 1:**
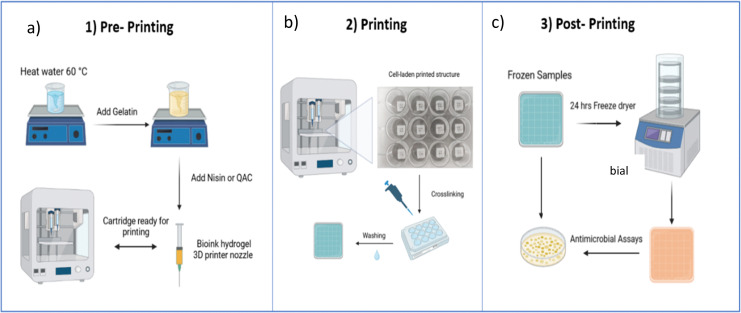
3D printing protocol and post processing of scaffolds. (a) Gelatin ink fabrication and cartridge preparation. (b) Printing and crosslinking of scaffolds. (c) Frozen samples can be used directly for antimicrobial assays or freeze-dried for future use in antimicrobial assays.

Gelatin (GL) inks were prepared at concentrations of 2.5, 5 and 10% w/w. These samples were denoted GL2.5 (2.5% gelatin), GL5 (5% gelatin), and GL10 (10% gelatin). Preparation of the inks is as follows: the GL5 ink was prepared as follows: 0.25 g of gelatin was added to 5 g of distilled water at 60 °C and stirred at 600 rpm until a visibly homogenous solution was acquired (1 h). For the nisin-loaded gelatin inks (GL-N), varying concentrations of nisin (1.125, 2.5, 5, 10 mg mL^−1^) were added to distilled water preheated at 50 °C and stirred at 600 rpm for one hour. The samples were denoted GL5-N1 (1.125 mg mL^−1^ nisin), GL5-N2 (2.5 mg mL^−1^ nisin), GL5-N5 (5 mg mL^−1^ nisin) and GL5-N10 (10 mg mL^−1^ nisin). The inks were loaded into 3 mL cartridges at room temperature 30 minutes before printing. The synthesis of the various inks is shown in Fig. 1(a). For the freeze-dried scaffolds, the gel scaffolds were frozen at −18 °C and lyophilized for 24 h in a freeze dryer (Scanvac CoolSafe Touch 110-4, Denmark). For the freeze dried (FGL) samples the same nomenclature as above was used. The nomenclature and conditions for all sample types are listed in Table 1.

**Table 1:** Nomenclature of non-freeze dried and freeze dried gelatin–nisin biomaterial ink composition

Biomaterial ink	Gelatin [%]	Nisin [mg mL^−1^]
GL2.5	2.5	—
GL5	5	—
GL10	10	—
GL5-N1	5	1.25
GL5-N2	5	2.5
GL5-N5	5	5
GL5-N10	5	10
FGL5	5	—
FGL5-N1	5	1.25
FGL5-N2	5	2.5
FGL5-N5	5	5
FGL5-N10	5	10

### 2.3. 3D printing

The structure of the 3D printed scaffolds was designed using 3D CAD drawing software (Creo Parametric®) to generate stereolithography (STL) files and converted to a G-code generator (Slic3r) which is integrated in Cellink's operating software (DNA®). During the slicing process, the layer height, printing speed and infill percentage were defined. The infill percentage is one of the most important features for 3D printing as it determines the pore size by defining the distance between two adjacent filaments. The 3D structures were printed by a dual printhead extrusion-based 3D printer (BIOX6, Cellink®) using a 27G nozzle at 28 °C with a pressure range between 100–115 kPa and a printing speed of 10 mm s^−1^. The final construct was made of twelve 0.2 mm layers, with an infill percentage of 20% giving a 2.5 mm distance between each adjacent filament. The scaffold dimensions were 10 × 10 × 2.5 mm. After printing, the scaffolds were crosslinked with a 0.5% glutaraldehyde solution for 30 minutes and left on a shaker at 50 rpm. The scaffolds were washed three times with water for five minutes each. Finally, the scaffolds were sterilized with UV light (265 m) for 30 minutes before use. A schematic illustration of the printing protocols is given in Fig. 1b. In order to enhance the durability and longevity of the scaffolds, freeze-drying was employed. Freeze-drying, also known as lyophilization, is a process wherein the scaffolds are subjected to low temperatures and reduced atmospheric pressure to remove moisture content. This meticulous dehydration method not only aids in preventing degradation but also contributes significantly to extending the shelf life of the scaffolds, ensuring their structural integrity and functional efficacy over an extended period. The scaffolds were frozen at −18 °C and lyophilized for 24 h in a freeze dryer (Scanvac CoolSafe Touch 110-4, Denmark).

### 2.4. Rheological analysis

The rheology of the inks was measured using an HTR 502 modular compact rheometer (Anton Paar, Ostfildern, Germany), using a 40 mm diameter parallel rough plate and a solvent trap to avoid evaporation of the hydrogel. The rough plate was lowered to a gap height of 1 mm and the excess ink was trimmed. Rheology of all samples were carried out in triplicate, and the results are represented as the average of three tests. A temperature sweep at 0.5% strain and 1 Hz was conducted by cooling the samples from 40 °C to 15 °C at a cooling rate of 1 °C min^−1^. Amplitude sweep tests were performed to identify the linear viscoelastic region of the samples in the range of 0.1–1000%, at a constant frequency (*f*) of 1 Hz. Frequency sweep tests were then performed at 0.5% strain, with a frequency range from 0.1–10 Hz. Complex viscosity (η*) was calculated from the storage (*G*′) and loss modulus (*G*′′) measured with the frequency sweep test. Measurements were performed at room temperature (21 °C). Dynamic mechanical analysis (DMA) was performed from the temperature sweep. tan delta (tan *δ*) was calculated to find the glass transition of the materials and characterize their viscoelastic behaviour.

### 2.5. Filament collapse test, diffusion rate and printability

The filament collapse test was conducted using the method published by Habib *et al.* (2019).^20^ An extruded filament is printed over a stage to evaluate its collapse by measuring its mid-span deflection. The stage was designed using Creo Parametric as shown in the ESI† (Fig. S1). The stage consisted of seven pillars with an incremental distance of 1 mm starting from 1 to 6 mm. The middle pillars dimensions were 2 × 10 × 6 mm. The dimensions of the two corner pillars are 5 × 10 × 6 mm. The design was printed using an Up Mini 23D printer with a polylactic acid (PLA) filament. The ink was printed as a single filament on top of the platform. A Sony IMX 682 64 MP 1/1.73′′ camera was used to immediately take a picture of the deposited filament to avoid time dependent deflection. The pressure, temperature, velocity of extrusion and nozzle diameter were the same as for printing the scaffolds. To determine the collapse area factor (*C*_f_) the following equation was used:^21–23^1
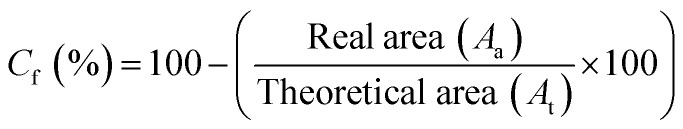
If the ink is not able to bridge between two pillars as a result of its density, the real area would be taken as zero, thus having a collapse area factor of a 100%. If the ink bridges between two pillars, the real and the theoretical area will be the same and the collapse area factor would be considered zero.

For the diffusion rate test, scaffolds with two consecutive layers were printed. The printed scaffolds followed a 0–90° pattern with increasing distance between adjacent filaments of 1–5 mm in 1 mm increments. To avoid time dependent deformation, images of the printed scaffolds were immediately taken with a Sony IMX 682 64 MP 1/1.73′′ camera after fabrication. The printing speed, temperature, pressure and nozzle diameter were the same as previously used. To calculate the diffusion rate (Df_r_) and printability (Pr) the following equations were used:^21–23^2
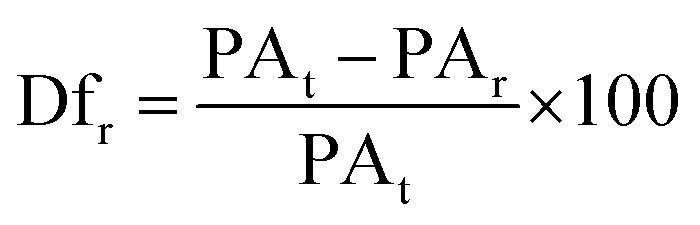
3
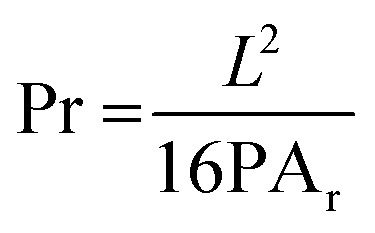
where PA_t_ and PA_r_ are the theoretical and real area of the grid spacing, respectively. *L* is the real perimeter of the grid spacing. The diffusion rate of the ink without any spreading is 0, while the printability would be 1 for a perfect square.

### 2.6. Shape fidelity in multi-layered structures

Samples were scanned with a Zeiss Xradia Versa 620 X-ray microscope, using a source accelerating voltage of 60 kV at 6.5 W, without a beam filter. The 0.4× objective was used to scan each whole sample with a voxel size of 19.25 microns (detector binning 2). For each scan 1601 projection images with an exposure time of 0.7 s each were collected over 360 degrees and reconstructed with Zeiss proprietary software Scout-and-Scan Reconstructor version 16.2. Reconstructed data were visualised using Drishti 3.2 software. Porosity and volume analysis were carried out using Fiji software.

### 2.7. Fourier-transform infrared spectroscopy (FTIR)

Attenuated reflectance FTIR was used to identify the chemical structure of the GEL and GEL-N scaffolds. The FTIR spectra were measured with a PerkinElmer Frontier IR Spectrometer (PerkinElmer, UK). The spectra represent an average of 32 scans recorded at a resolution of 4 cm^−1^ in the range from 600–4000 cm^−1^.

### 2.8. Morphological features (SEM) and porosity

The morphological features of the scaffold were analyzed by scanning electron microscopy (JEOL, JSM-7001F, analytical scanning electron microscope, Tokyo, Japan) with accelerated voltages of 10 kV. The samples were sputter coated (Q150T ES) with a gold layer. Pore size was determined by analyzing the SEM images with ImageJ software.

### 2.9. Swelling ratio of the hydrogel

The swelling performance of the scaffolds was determined by immersing the samples in sterile PBS at 37 °C for 72 h measuring the sample every 6 hours. The swelling rate (Sw%) was calculated with the following formula:^21–23^4
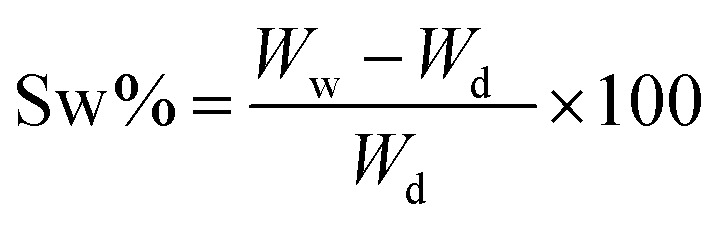
where *W*_w_ and *W*_d_ denote weight in the swollen and dry state. Excess water was first absorbed with laboratory paper towels. Measurements were done in triplicate.

### 2.10. Degradation

To calculate the degradation rate, the samples were immersed in PBS and incubated at 37 °C. The scaffolds were then removed every 6 hours up to 72 hours. The scaffolds were dried and weighed, and the remaining weight of the scaffold after degradation was calculated using the following equation:^21–23^5
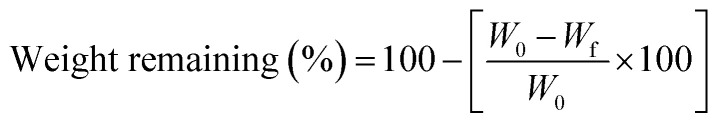
where *W*_0_ is the initial weight of the scaffold and *W*_f_ is the final weight of the scaffold.

### 2.11. Nisin release

The nisin release from the scaffold was determined by high-performance liquid chromatography (HPLC) using the Agilent Technologies 1260 Infinity II System (Radnor, USA) under isocratic conditions at room temperature and equipped with a 4.6 × 250 mm ZORBAX SB-C18 analytical column. To determine the release profile, all scaffolds were analyzed under the same conditions. Multiple scaffolds were printed and submerged in 1 mL of PBS at 37 °C for different time points. Then, each aliquot was centrifuged at 4000 × *g* for 15 minutes, and any undissolved gelatin/peptide was filtered through the protein concentrator. A mixture of acetonitrile (90% in 0.1% formic acid) and water (10%) was used as the mobile phase, at a flow rate of 1 mL min^−1^, with UV detection at 254 nm and an 8 nm bandwidth. For the HPLC samples, an injection volume of 50 μL, handled by a (G7129A) autosampler, was used. Quantification of the concentrations was determined by the peak area.

### 2.12. Antimicrobial assay (planktonic)

Overnight cultures of methicillin-resistant *Staphylococcus aureus* (*MRSA*) NCTC 13142 and *Pseudomonas aeruginosa* (*P. aeruginosa*) NCTC 07244 were prepared by statically incubating *MRSA* in nutrient broth and *P. aeruginosa* in Luria-Bertani (LB) at 37 °C for 24 hours. The resulting cultures were diluted to a cell concentration of 10^6^ colony forming units per millilitre (CFU mL^−1^) using a 0.5 McFarland Standard.^24^ 3D printed scaffolds were inoculated with 2 mL of the diluted cultures and incubated in a shaking incubator at 37.0 °C for 4 and 24 hours. At each time point, the incubated samples were serially diluted and plated on NB agar plates (for *MRSA*) or LB agar plates (for *P. aeruginosa*) using the Miles and Misra method^25^ to determine the CFU of each bacterial suspension. The antimicrobial efficiency of the bioprinted scaffolds was evaluated based on the reduction in CFU over time.

### 2.13. Biofilm inhibition assay

For the biofilm inhibition assay, samples were inoculated with the diluted cultures following the same procedure described for the planktonic assays. The inoculated samples were incubated in a shaking incubator at 37.0 °C for 24 hours. Following incubation, the samples were gently rinsed with phosphate-buffered saline (PBS) to remove non-adherent bacteria. Subsequently, the samples were sonicated for 15 minutes in either 1 mL of LB or NB broth to detach and resuspend the biofilms. The bacterial colony-forming units were assessed after serial dilution of the bacterial suspension using the Miles and Misra^25^ technique on LB agar plates.

### 2.14. Nisin stability

The stability of nisin was evaluated against *MRSA* and *Pseudomonas aeruginosa* at 1, 2, 3, and 4 weeks. The antimicrobial efficacy of the gel and freeze-dried samples were tested at 4 and 24 hours. Only the scaffolds with the highest concentration of nisin (GL5-N10 and FGL5-N10) were tested for their stability.

### 2.15. Statistical analysis

One-way analysis of variance (ANOVA) was used to compare whether the difference in antimicrobial efficiency and other measurements between each bioprinted sample was significantly different. A value of *p* < 0.05 was taken as being statistically significant.

## 3. Results and discussion

An antimicrobial biomaterial ink for 3D printing should have appropriate properties including printability, shape fidelity after printing, mechanical properties (or stability), and biological properties (including degradation/insolubility in physiological solutions).^12,21–23,25–27^ In terms of printability, the ink should have shear-thinning properties such that viscosity assists printing during extrusion, and the ink should have high recoverability properties such that the initial viscosity post printing is regained for the construct to be stable.^23^ The ink should have the appropriate mechanical properties to self-support and spread the printed ink on surfaces and provide support for the top printed layers.^23^ Moreover, the printed scaffold should be able to store the appropriate payload of the antimicrobial to be released at the rate and concentration required. All of these properties have been comprehensively evaluated through the course of this study.

### 3.1. Rheology

**Fig. 2:**
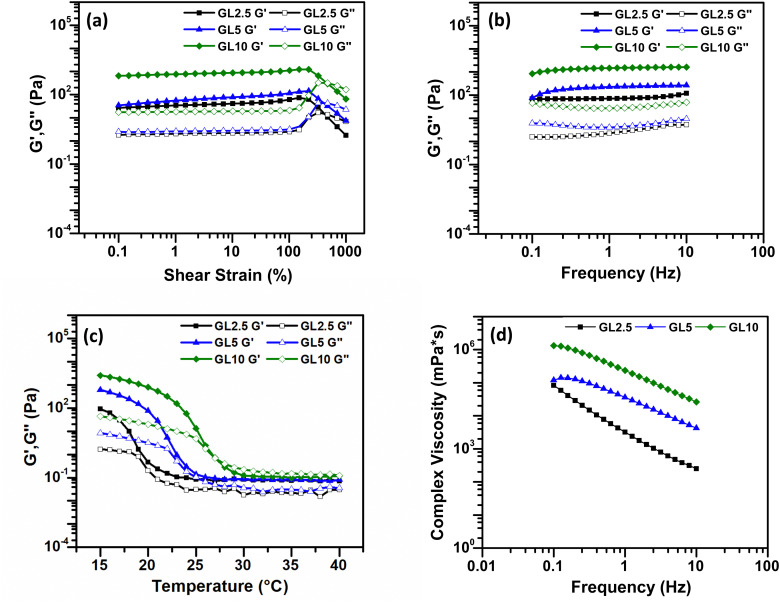
Rheological analysis of gelatin bioinks. (a) Amplitude sweep test. (b) Frequency sweep test. (c) Temperature dependence sweep. (d) Viscosity as a function of frequency. *N* = 3.

To comprehensively understand the fundamental behaviours of gelatin (GL) and gelatin-nisin (GL-N) biomaterial inks, a thorough investigation of their rheological characteristics was conducted before the printing process using oscillatory shear. The rheology in terms of the strain amplitude response, frequency dependence in the linear viscoelastic regime (*i.e.* small amplitude oscillatory shear limit), temperature profile and complex viscosity are measured and the results are shown in Fig. 2 for the pure gelatin inks (GL2.5, GL5, GL10) and Fig. 4 for the gelatin-nisin (GL5-N1, GL5-N2, GL5-N5, GL5-N10) inks.

For the pure gelatin biomaterial inks, amplitude sweep analyses were carried out at room temperature (21 °C) over a strain range of 0.1–1000% to measure the viscoelastic properties (Fig. 2(a)). All three inks displayed viscoelastic properties that extended beyond 100% strain, indicating the biomaterial ink's ability to undergo significant deformation without structural failure or loss of integrity.

The storage modulus *G*′, represents the elastic or solid-like property of the material (for a simple viscous Newtonian liquid this is identically zero). Whereas the so-called loss modulus *G*′′, represents the viscous component of the material (for a perfectly Hookean elastic solid this is identically zero). To achieve extrusion-based printability, the solid properties of biomaterial inks are not weaker than the liquid properties, that is, the storage modulus, *G*′, should be equal to or even higher than the loss modulus, *G*′′ to ensure the formation of 3D structures. From the frequency sweep test, conducted at a constant strain of 0.5% within the linear viscoelastic region, both *G*′ and *G*′′ were measured. For all three inks, a *G*′ greater than *G*′′ indicated a more gel-like character, and the gel strength exhibited an increase with higher concentration. The plateau moduli observed demonstrated the stability of all the inks (Fig. 2(b)).

Subsequently, temperature sweep analysis (Fig. 2c) was used to understand the temperature-dependent behaviour of the biomaterial inks. This is an important parameter to characterize as it will provide useful information regarding controlled extrusion and shape fidelity. For the controlled extrusion, the gelatin ink viscosity depends heavily on temperature. Characterizing these changes allows optimal printing temperature selection. At higher temperatures, the ink is less viscous and flows easily through the printer nozzle. As temperature drops, the ink thickens, potentially causing extrusion difficulties or inconsistencies. For the shape fidelity, understanding the ink's viscosity at different temperatures helps predict and control the printed structure's shape. Higher viscosity at lower temperatures can lead to better shape retention, while lower viscosity at higher temperatures allows for finer details and overhangs.

In Fig. 2c, as the temperature gradually decreased and approached the gelation temperature (20–30 °C), both *G*′ and *G*′′ increased rapidly due to the gelation process, also called the sol–gel transition. Gelatin is a hydrolytic derivative of collagen that is widely used in tissue engineering. Gelatin dissolves in solutions when the temperature is above 50 °C, and it will reversibly form an α-helical structure when the temperature drops to below 30 °C.^24^ A one percent gelatin aqueous solution will produce chain association and a 3D network and the reversibility of this helical structure depends on the concentration of gelatin and solution temperature.^28^ The temperature-sensitive phase transition property of gelatin is key to maintaining the 3D structure of printing at a certain printing temperature.

Finally, the complex viscosity can be determined from the storage and loss moduli. Often this quantity can be a good proxy for the shear viscosity that is determined in steady-shear flow (recall here that our measurements are obtained in oscillatory shear). The viscosity curve illustrated the variation in complex viscosity (η*) with changing frequency (*f*) (Fig. 2(d)). The higher concentrations of gelatin within the inks correlated with increased viscosity, while all inks’ viscosities decreased at higher frequencies. Gelatin, a protein-based substance, serves as a thickening agent in inks, controlling their viscosity (the resistance to flow). When gelatin concentration increases in the ink's formulation, there is a greater abundance of gelatin relative to other ink components. Gelatin molecules become entangled, creating interactions that impede the flow of the ink. As a result, higher concentrations of gelatin lead to increased viscosity, resulting in thicker, more viscous inks.^29^

A decrease in viscosity at higher frequencies is observed. This viscosity reduction can be attributed to the disruption of gelatin networks. As a consequence, the ink becomes more fluid and flows more readily.^30^ This is indicative of the shear-thinning nature of the inks, which is a crucial characteristic for extrusion-based printing applications. Shear-thinning materials have the ability to undergo transient structural reconfiguration when exposed to applied pressure and quickly reassemble after removal of the pressure and resulting shear. This could be due to the stretching or possible (partial) alignment of the polymer chains in the pure gelatin inks. The dynamic nature of these interactions between the molecules can be disrupted for a short time under shear, but reforms once again upon removal.^31^ According to the rheological findings, all concentrations of gelatin demonstrate promising suitability for 3D printing, exhibiting shear-thinning behaviour, solid-like characteristics at room temperature, and frequency independence for both storage and loss modulus, although only observed for GL5 and GL10. Generally, the success of printing with an ink relies on its viscosity. Viscosities greater than 30 mPa s are suitable for extrusion-based bioprinting.^32^ The extrusion of biomaterial inks is a process of applying shear force, and the rheology and viscoelasticity of biomaterials affect its printability.^33^ The rheological outcomes across GL2.5, GL5, and GL10 prompted further characterization to determine efficient printability and shape fidelity (refer to Section 3.2). Consequently, GL5 was selected for the incorporation of nisin, given its superior qualitative and quantitative results in terms of both printability and diffusion rate percentage. GL2.5 exhibits an initial sharp increase in tan delta around 19 °C, suggesting a rapid transition from a predominantly elastic to a more viscous state. While tan delta fluctuates after peaking near 23 °C, it generally maintains an elevated level compared to its initial value. GL5 demonstrates a steady increase in tan delta, culminating in a peak around 24 °C. Afterwards, tan delta undergoes minor fluctuations but persists at a relatively high level, indicative of a continuous rise in viscous behaviour. GL10 displays a pronounced viscoelastic transition, characterized by a substantial increase in tan delta commencing around 25 °C. This is accompanied by a prominent peak (glass transition) and subsequent fluctuations, signifying a dominant viscous response at elevated temperatures. At lower temperatures, GL2.5 exhibits greater elasticity, transitioning to a more viscous state near 19 °C. However, this transition is less pronounced compared to GL10. GL5 undergoes a more gradual shift in viscosity, ultimately displaying less viscous characteristics than GL10 at higher temperatures. Following the initial transition, GL2.5 demonstrates more stable tan δ values, while GL5 and GL10 continue to exhibit variations at elevated temperatures (see the ESI,† Fig. S2).

**Fig. 3:**
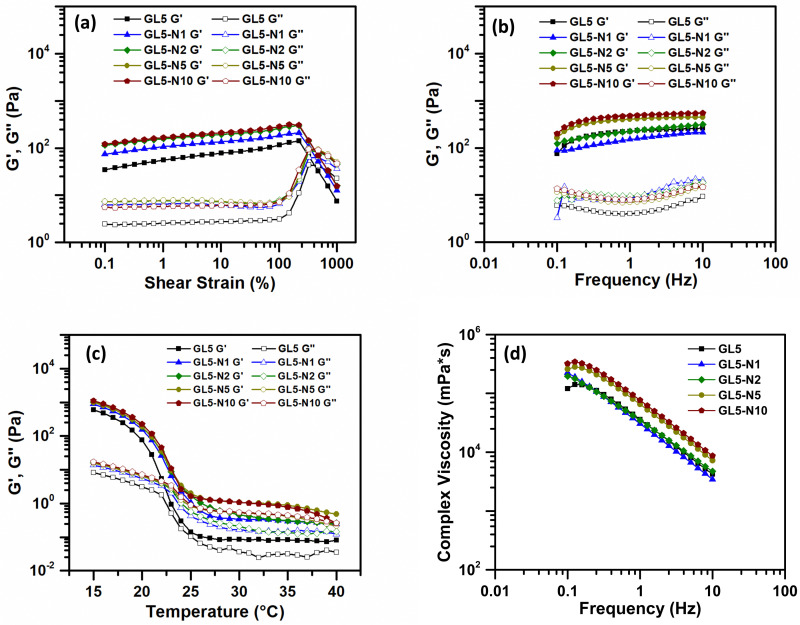
Rheological analysis of gelatin + nisin bioinks. (a) Amplitude sweep test. (b) Frequency sweep test. (c) Temperature dependence sweep. (d) Viscosity as a function of frequency. *N* = 3.

Nisin was incorporated into the GL5 ink at various concentrations, namely GL5-N1, GL5-N2, GL5-N5, and GL5-N10, and the rheological behaviour of these inks was studied. Amplitude sweep analyses conducted at room temperature (21 °C) across a strain range of 0.1–1000% (Fig. 3(a)) revealed that all four inks exhibited an approximate linear viscoelastic region extending beyond 100% strain. This behaviour mirrored that of the GL5 ink without nisin. The consistent behaviour observed across all nisin concentrations suggests that the addition of nisin does not significantly alter the rheological properties of the ink, including its viscoelasticity, frequency and temperature dependence, and viscosity, when compared to GL5. Statistical analysis further confirmed that there were no significant differences in any rheological parameter between the nisin inks and the GL5 sample. This observation extends to the impact the addition of nisin would have on the material properties, such as the plateau moduli in the frequency sweep, indicating the stability of all GL5-N inks (Fig. 3(b)), regardless of nisin content.

In the temperature sweep, a temperature-dependent behaviour was observed across all GL5 and GL5-N samples. As the temperature gradually decreased, both *G*′ and *G*′′ increased rapidly for GL5-N1, GL5-2 and GL-N5, similar to the GL5 sample. Finally, viscosity measurements demonstrated that the shear-thinning nature of the inks was consistent in all GL5 and GL-N samples. There were no statistically significant differences observed in the viscosities. This finding emphasises the robustness of the shear-thinning behaviour across all formulations, suggesting that, despite variations in nisin concentration, the viscosities remained statistically comparable across the GL5-N*x* samples. This shear thinning behaviour was also observed by Luciano *et al.*, 2020, who observed no significant effect on the flow curves for nisin loaded gelatin films indicating there was no strong interaction between the nisin and the polymeric matrix that could change the behaviour of the gelatin film solutions.^20^

### 3.2. Filament collapse test, diffusion rate and printability

A filament collapse test was conducted to assess the feasibility of continuously extruding or printing the GL2.5, GL5, GL10 and GL5-N1, GL5-N2, GL5-N5 and GL5-N10 inks in the absence of external support structures. The objective was to determine whether the printed materials could retain their shape and integrity. Fig. 5 illustrates the experiment, where the inks were printed over a series of seven pillars arranged in a linear array, gradually spaced apart by 1–6 mm.^23^ This assesses the collapse area factor of a biomaterial ink, where the ink's density affects its ability to form a bridge between two pillars. In this instance, where the ink's density is ineffective at bridging, the real area is regarded as zero, and the collapse area factor as 100%. Conversely, if the ink successfully bridges the gap between pillars, aligning the real and theoretical areas, the collapse area factor is deemed to be zero. This metric becomes a pivotal measure in classifying the structural integrity and performance of the biomaterial inks, which in turn provides insights into their ability to maintain and support 3D printing. The collapse area factor thus serves as a valuable parameter in evaluating the practical utility and robustness of inks in diverse bioprinting contexts.^34,35^

**Fig. 4:**
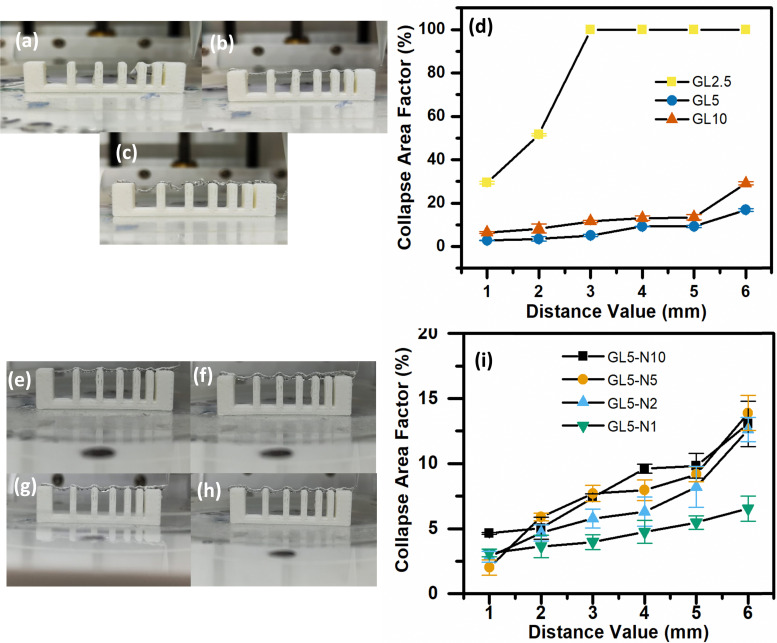
Filament collapse test for gelatin bioinks. (a) GL2.5 ink, (b) GL5 ink, and (c) GL10 ink. (d) Collapse area factor as a function of distance for all gelatin bioinks. Filament collapse test for gelatin/nisin bioinks. (e) GL5-N1 ink and (f) GL5-N2 ink. (g) GL5-N5 ink and (h) GL5-N10 ink. (i) Collapse area factor as a function of distance for all gelatin/nisin bioinks.

For the pure gelatin inks, GL2.5 failed to maintain its integrity beyond pillars separated by 1 and 2 mm, and had a collapsed area factor of 100% beyond 3 mm separation (Fig. 4(a)). GL5 formed almost straight filaments, which did not bend as the gap between pillars was increased (Fig. 4(b)). The GL10 ink was able to bridge between all separations, but failed to make smooth filaments, with several bumps along the filament, and a higher collapsed area factor for the higher separations when compared to the GL5 ink (Fig. 4(c)). Overall, the results showed the GL5 ink to have a lower collapse area factor along the separations gaps (Fig. 4(d)).

For the GL-N inks, the highest concentration of nisin, GL-N10, resulted in a higher percentage of collapsed area factor. This finding suggests that as the concentration of nisin increased, the collapsed area factor increased (Fig. 4(i)). One possible explanation for this observation is the relative reduction of gelatin in proportion to the total weight of the nisin/gelatin ink when compared to the 5G gelatin ink. This was also seen by Luciano *et al.*, 2020, by demonstrating that the increase of nisin in their gelatin solutions reduced both the tensile strength and elastic modulus by a factor of 2 by every 50 mg of nisin added to the solution.^20^

When evaluating the performance of the inks, it was found that all the inks were capable of bridging the gaps across the platforms effectively. This indicates that regardless of the concentration of nisin, the inks exhibited a low collapse area factor (<20%) (Fig. 4(e–i)). However, the addition of higher amounts of nisin resulted in an increased collapsed area factor. This could be attributed to the alteration of the ink's physical properties due to the higher concentration of nisin. The concentration of gelatin plays a crucial role in determining the overall behaviour of the ink. Gelatin, being a hydrogel-forming material, contributes to the structural integrity and stability of the printed scaffolds. Therefore, when the relative proportion of gelatin decreases in relation to the nisin content, the ink's ability to maintain its shape and prevent collapsing may be compromised.^20^ This could explain why higher concentrations of nisin led to a higher collapsed area factor. It was observed that concentrations below 2.5 wt% exhibit significantly diminished printability, failing to produce satisfactory results in terms of structural integrity and overall quality. As such, 2.5 wt% effectively serves as the lower threshold for practical application, ensuring that our material maintains the necessary properties for successful printing. Conversely, while concentrations above 10 wt% may still be printable, this upper limit was set to ensure a balance between ease of printing and material performance, thus defining a comprehensive range for effective use.

**Fig. 5:**
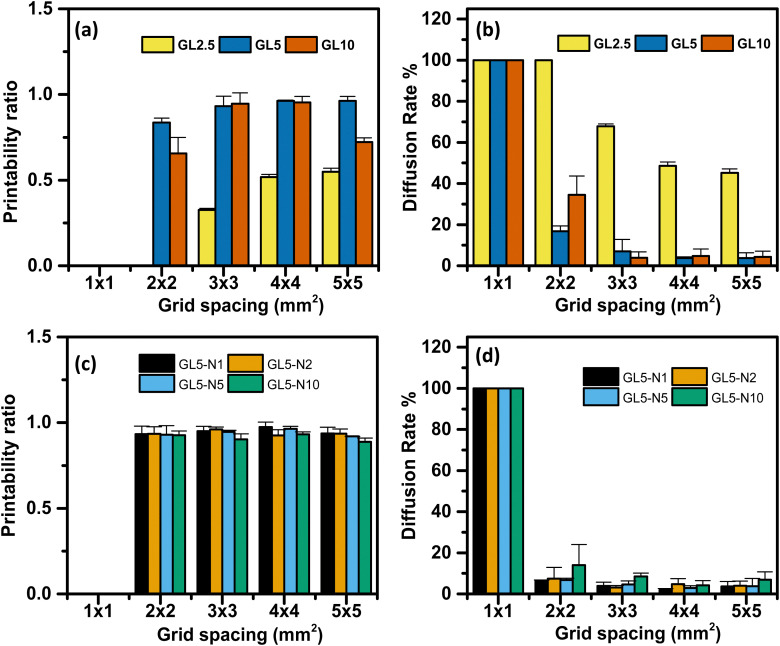
Printability ratio and diffusion rate percentage of the GL2.5, GL5, GL10, GL5-N1, GL5-N2, GL5-N5, and GL5-N10 inks. (a) Printability ratio of the GL2.5, GL5 and GL10 inks. (b) Diffusion rate % of the GL2.5, GL5 and GL10 inks. (c) Printability, GL5-N1, GL5-N2, GL5-N5, and GL5-N10 inks. (d) Diffusion rate % of GL5-N1, GL5-N2, GL5-N5, and GL5-N10 inks.

The influence of filament diffusion and printability of each ink was assessed using eqn (2) and (3). Qualitatively, it was observed that the 5G and 10G inks exhibited favourable grid spacing geometry and minimal material diffusion compared to the 2.5G ink (Fig. 5(a–c)). Moreover, as the grid spacing size increased, the printability of the inks also improved. These qualitative findings were further supported by quantitative analysis, which confirmed that the 5G ink demonstrated the overall best performance.

Regarding the diffusion rate, it was found that the smallest grid spacing size exhibited the highest diffusion rate and lowest printability across all the ink ranges. This highlights the importance of achieving a minimum resolution higher than 1 mm to optimize the performance of these inks. In the case of the nisin/gelatin range, there were no significant differences in the diffusion rate and printability between the inks, indicating that the addition of nisin did not significantly impact the shape fidelity and printability of the inks (Fig. 5(d)). This suggests that the incorporation of nisin into the gelatin matrix does not lead to drastic changes in the overall performance of the inks.

To conclude, the qualitative and quantitative analysis revealed that the grid spacing geometry, diffusion rate, and printability of the inks were influenced by the composition and grid spacing size. GL5 ink exhibited superior performance, while the smallest grid spacing size demonstrated the highest diffusion rate and lowest printability.

Additionally, the inclusion of nisin in the gelatin matrix had minimal effects on the diffusion rate and printability of the inks. The excellent printability of the 5% gelatin (GL5) formulation can be seen by the outcomes of a diffusion rate percentage experiment designed to evaluate the printed shape fidelity of 3D scaffolds (see Fig. S4, ESI†). Scaffolds were fabricated using a range of gelatin concentrations, specifically 2.5%, 5%, and 10% w/w. The results demonstrate that the GL5 formulation achieved optimal shape fidelity, characterized by superior resolution and a notable absence of structural defects. In contrast, the 10% gelatin exhibited diminished shape accuracy, likely due to the increased viscosity hindering the extrusion process. Conversely, the 2.5% gelatin formulation proved inadequate in maintaining filament integrity, suggesting insufficient material viscosity for robust scaffold formation. GL5 displaying similar printability with other gelatin based inks has been observed by Serafin *et al.*, where a gelatin hydrogel with varying polypyrrole nanoparticles was used to print 10-mm square lattices.^36^ All samples demonstrated good shape fidelity with defined filament lines and maintained height, and they could be handled at room temperature without crosslinking.

### 3.3. Shape fidelity in multi-layered structures (micro-CT)

**Fig. 6:**
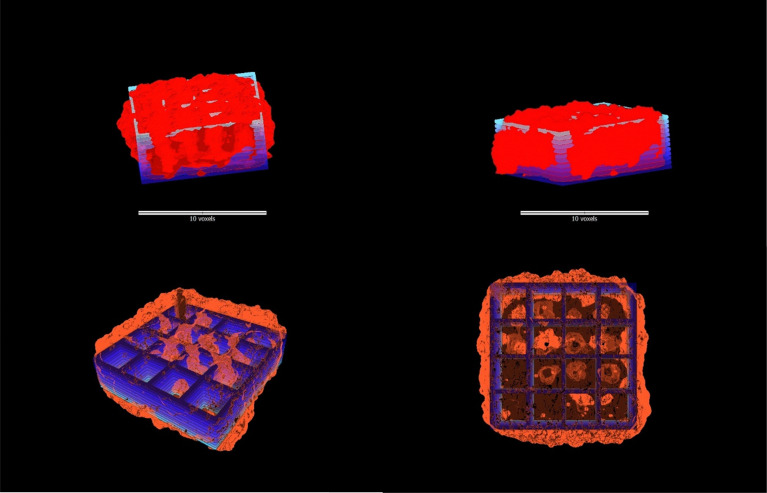
Overlay of the CAD design scaffold (blue) with micro-CT images (red) demonstrating the scaffold architecture and multi-layered structural fidelity.

Computer-aided design (CAD) of a multilayer grid was utilized to fabricate a 3D-printed scaffold, which was subsequently analysed using micro-computed tomography (micro-CT) imaging. By overlaying the digital CAD model with the reconstructed 3D image, a detailed shape comparison was conducted (Fig. 6). The analysis indicates the excellent shape retention of the printed scaffold, as evidenced by the close alignment and congruence between the designed and fabricated structures in terms of layer stacking and structural integrity. The overall shape of the scaffold is well-preserved, with both the printed scaffold and the CAD design exhibiting a similar rectangular structure with internal supports. However, minor deviations were observed in the positioning of the internal features, with the printed scaffold showing slightly more variation in the arrangement of these structures. The bottom layer of the scaffold also demonstrated good shape retention, with both the printed scaffold and the CAD design displaying a similar grid-like pattern. Notably, the printed scaffold exhibited some additional swelling not present in the CAD design. This discrepancy could be attributed to the post-printing freezing process employed to preserve the scaffold for an extended period.

### 3.4. Fourier-transform infrared spectroscopy (FTIR)

FTIR was used to confirm the presence of gelatin and nisin within the scaffolds. Three scaffolds were assessed: GL5, GL5-N1 and GL5-N10. Since the chemical structure of gelatin and nisin (functional groups) is constituted of amino acid residues, there are similar peaks observed in their FTIR spectra.^37^ The main signal at 3290 cm^−1^ is amide A, corresponding to the N–H stretching overlapping the O–H stretching vibration. The amide I band at 1650 cm^−1^ represents C=O stretching of protein amide groups. The peak at 1552 cm^−1^, amide II, is the N–H stretching of protein.^37^ FTIR was not able to confirm the presence of nisin within the scaffolds given the overlap in the peaks (see the ESI,† Fig. S3).

### 3.5. Scanning electron microscopy (SEM)

**Fig. 7:**
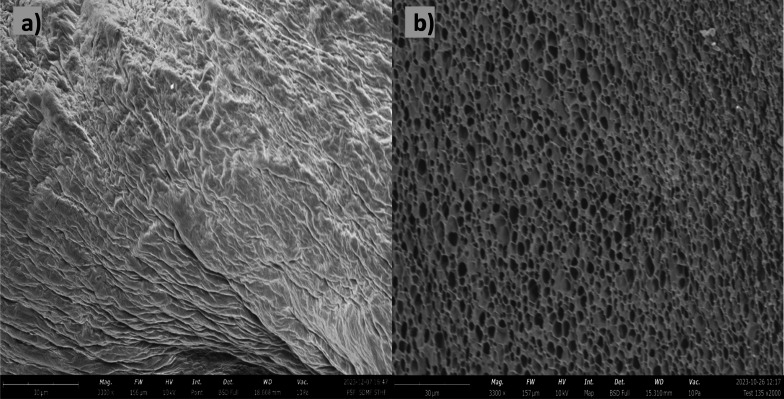
Scanning electron micrographs of the (a) non-freeze-dried scaffold, and (b) freeze dried scaffold.

The evaluation of porosity in the scaffold was conducted through SEM imaging, and subsequent image processing was carried out utilizing ImageJ for enhanced accuracy. Fig. 7(a) shows the SEM image of the non-freeze-dried scaffolds, while Fig. 7(b) shows the freeze-dried scaffold. The non-freeze-dried scaffold displayed a seemingly homogeneous surface without discernible pores, suggesting a dense and uniform structure. Although it is important to note that directly imaging a gelatin gel in its wet form using traditional SEM is not truly representative. This is because SEM requires samples to be in a vacuum and under high voltage, which would cause the wet gel to evaporate and collapse, making it hard to capture its structure accurately.

In contrast, the freeze-dried version exhibited distinctive pores scattered across its surface, indicative of the impact of freeze-drying. The calculated average pore size is reported at 0.691 μm^2^. Moreover, the porosity at the microscale is determined to be 93% within a scanned area spanning 1600 μm^2^. This examination at the micro level underscores the precision of the fabrication process and highlights the scaffold's capacity for accommodating highly porous and intricate microstructures in its freeze-dried form. This has been observed by Liu *et al.*, were the pore formation in the gelatin hydrogels was due to the freeze-drying process.^13^ Azizian *et al.*, investigated the impact of different freezing temperatures on the porosity and pore size of chitosan/gelatin freeze-dried scaffolds.^38^ The scaffolds’ porosity increased with decreasing freezing temperature, as lower temperatures led to the formation of larger ice crystals, resulting in larger pores after sublimation. Pore size distribution also varied with freezing temperature, with lower temperatures yielding scaffolds with a wider range of pore sizes, including both larger and smaller pores. SEM images confirmed the presence of interconnected pores within the scaffolds.

### 3.6. Swelling and degradation

**Fig. 8:**
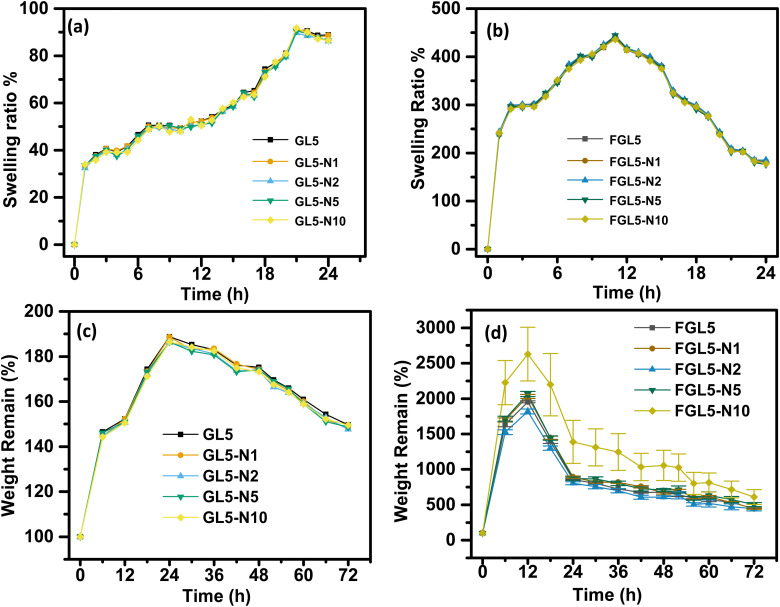
(a) Swelling ratio of the gel scaffolds over a 24-hour period. (b) Swelling ratio of the freeze-dried scaffolds over a 24-hour period. (c) Degradation percentage of the gel scaffolds over a 72-hour period. (d) Degradation percentage of the freeze-dried scaffolds over a 72-hour period.

The swelling behaviour of the scaffold in water over a 24-hour period is shown in Fig. 8. All scaffolds, non-freeze dried (Fig. 8a) and freeze dried (Fig. 8b) had a burst swelling period within the first hour of suspension in water. This initial burst of water uptake is most likely caused by the hydrophilic nature of the polymers that quickly absorb water.^39^ This burst period was followed by a slower increase in swelling followed by a quicker swelling rate until maximum swelling was reached at 20 h for the non-freeze-dried scaffold and 12 h for freeze dried scaffolds. This lower swelling rate could be due to a delay in water penetrating the scaffolds, which has been attributed to the microporous architecture structure by Gupta *et al.*, 2021.^39^ Once the water penetrated the 3D microarchitecture, the swelling rate increased again due to the surface tension and capillary action, as the micropores had already been infiltrated with water.

A significantly higher increase in swelling ratio of the FGL5, FGL5-N1, FGL5-N2, FGL-N5, and FGL5-N10 samples was observed compared to that of the GL5, GL5-N1, GL5-N2, GL5-N5 and GL5-N10 samples. The freeze-dried scaffolds showed an increase of 350% between the initial measurement and the second measurement, whereas the gel scaffolds increased their swelling ratio by only 35%. The overall degree of swelling depends on the number of free non-crosslinked polymeric chains as well as on the micro-architecture of the scaffold.^37,38^ This difference can be attributed to the initial water content. The freeze-dried scaffold starts in a dehydrated state with more available space to absorb a solvent, while the gel form is already saturated with water, leaving less capacity for additional swelling.^40^ There was no observed difference in the swelling behaviour with and without nisin regardless of the concentration of nisin.

This difference in behaviour is linked to the freeze-dried scaffold's heightened porosity and increased surface area, which facilitates more rapid water penetration. Consequently, this accelerated penetration leads to an elevated rate of degradation.^41^ The degradation study was carried out over a 72-hour period in PBS and the results are displayed in Fig. 8(c) for the non-freeze dried samples and Fig. 8(d) for the freeze-dried samples. Degradation of the non-freeze-dried samples began after 24 h and they degraded about 20% over a 72 h period.

The freeze-dried scaffold started to degrade after 12 h and degraded to 80% over a 72 h period. The increase in the degradation rate of the freeze-dried scaffolds can be attributed to the greater porosity and increased surface area. This has also been observed by Zhang *et al.*, for the effect of porosity in PCL scaffolds.^41^ It was observed that an increase in porosity resulted in an increase in water uptake and an increase in the degradation rate. During the degradation process, water infiltrated the material, triggering hydrolysis and random chain fragmentation. Subsequently, the segmented chains diffused out of the material, resulting in a reduction in weight.^41^ Furthermore, the disparity between the degradation rate of the non-freeze dried and freeze-dried samples can be attributed to pore size distribution and interconnectivity as also observed by Gupta *et al.* for 3D printed gelatin-gellan gum scaffolds.^39^ The scaffold fabricated in their study showed an increase in micropores, nanoscale functionality and interconnectivity, achieving a hierarchical porous structure with freeze drying. Degradation of the gelatin scaffolds occurs by acid or base catalysed amide bond hydrolysis generating degradation products and H^+^ and OH^−^ ions which can further autocatalyze the degradation reaction.^42^ The degradation rate escalates when the removal rate (clearing from the pore wall) of degradation products lags behind their production rate, leading to the accumulation of degraded products. In the case of freeze-dried scaffolds, enhanced interconnectivity, thinner pore walls, and increased surface area contribute to a more efficient removal of degradation products, resulting in a slower degradation rate after 24 hours.^43^ Between 12 and 24 hours, there is a potential accumulation of degradation products, which may contribute to an increase in degradation. Conversely, non-freeze-dried samples with thicker walls and lower pore interconnectivity tend to accumulate degradation products, leading to a faster degradation rate after 24 hours.^43^

### 3.7. Nisin release

**Fig. 9:**
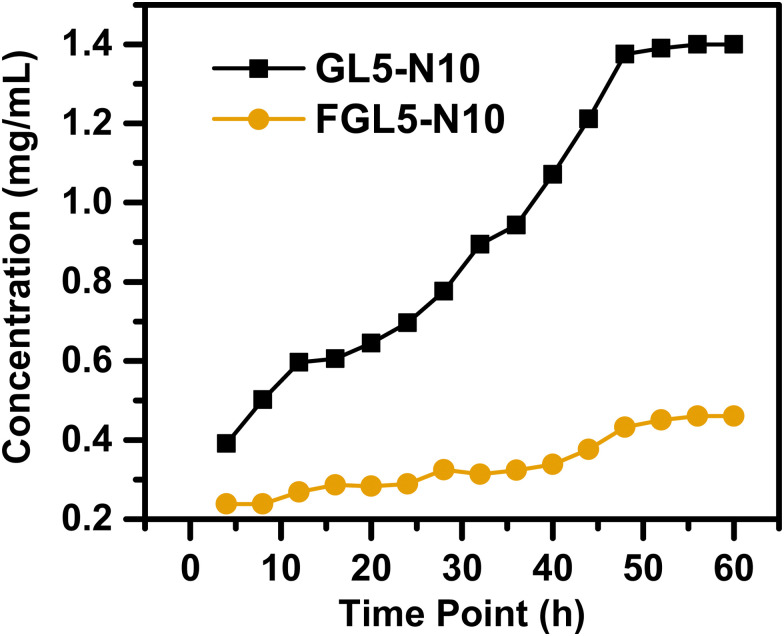
Nisin drug release over a 60-hour period measured by high-performance liquid chromatography (HPLC).

The nisin release from the scaffold was determined using high-performance liquid chromatography (HPLC) and the results are given in Fig. 9. To establish the release profile, the scaffolds GL5-N10 and FGL5-N10, with the highest nisin concentration (*i.e.*, 10 mg mL^−1^) were analysed. The nisin release profile of the lowest concentrations can be found in the ESI† (Fig. S5).

For the nisin release, both the non-freeze dried and the freeze-dried scaffolds showed an increased release with time (Fig. 9). At 4 h, the non-freeze-dried scaffold (GL5-N10) released 0.4 mg mL^−1^ and the freeze-dried scaffold (FGL5-N10) released 0.2 mg mL^−1^. GL5-N10 showed a higher released concentration of nisin compared to FGL5-N10. At 48 h, GL5-N10 released 1.4 mg mL^−1^, compared to FGL5-N10 release which was 0.4 mg mL^−1^.

In addition, FGL5-N10 showed a steadier rate of release. This was also observed by Shalaby *et al.*, where after 90 minutes of swelling the scaffolds in their chosen media, about 63.7% (by weight) of the total loaded drug was released from gels that were not freeze dried.^44^ In contrast, gels that were freeze-dried released only an average of 38.6% (by weight) of the total loaded drug during the same period. They hypothesized this was due to drug uniformity dispersion in the freeze-dried gels compared to the non-freeze-dried gels where high amounts of the drug localized at the surface.^44^ In the freeze-dried sample, although less nisin is incorporated into the gels, due to being potentially washed away by the freeze-drying process, the remaining nisin may be present in the correct orientation to have an antimicrobial effect. In this vein, Aveyard *et al.* showed that the N-terminus of the nisin peptide needs to be available for interaction and disruption of the bacterial cell wall.^45^

### 3.8. Antimicrobial assay (planktonic)

**Fig. 10:**
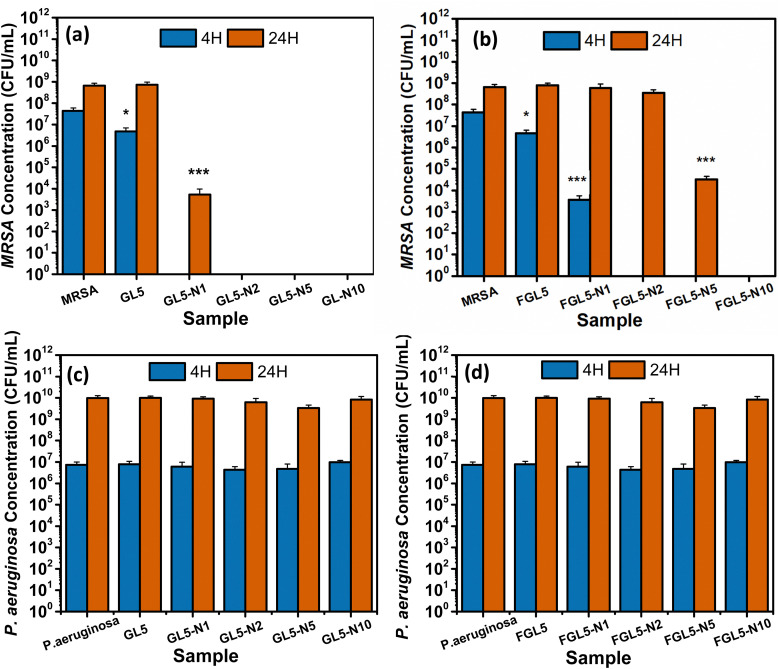
Planktonic inhibition assay of nisin scaffolds in gel and freeze-dried form at 4- and 24-h time points. (a) Antimicrobial efficacy of the non-freeze-dried scaffolds against *MRSA*. (b) Antimicrobial efficacy of the freeze-dried scaffolds against *MRSA*. (c) Antimicrobial efficacy of the non-freeze-dried scaffolds against *Pseudomonas aeruginosa*. (d) Antimicrobial efficacy of the freeze-dried scaffolds against *Pseudomonas aeruginosa*. *P < 0.05, **P < 0.01, ***P < 0.001 (*N* = *3*, *n* = *3*).

The antimicrobial effectiveness of nisin-loaded scaffolds was assessed against *MRSA* and *Pseudomonas aeruginosa* at 4 and 24-hour time periods and the results are shown in Fig. 10. For both the non-freeze dried and freeze-dried scaffolds with added nisin there was no bacterial colonisation observed at 4 hours, except for the lowest concentration, FGL5-N1 in the freeze-dried scaffold (Fig. 12(b)). After 24 hours, all non-freeze-dried scaffolds displayed complete bacteria killing except for the FGL5-N1 sample which displayed a 6-log reduction as seen in Fig. 10(a). After 24 hours, only the FGL5-N10 sample demonstrated complete killing of MRSA. FGL-N5 showed a 5-log reduction, while FGL5-N2 and FGL5-N1 demonstrated no reduction to the bacterial counts (Fig. 10(b)).

There was no antimicrobial activity observed against. *P. aeruginosa*. This is as expected as nisin only has activity against Gram positive bacteria.^43^

These results align with expectations as nisin's efficacy typically increases with higher concentrations, as reported by Jensen *et al.*^18^ The mechanism of action of nisin involves its interaction with the lipid II precursor, which is vital for peptidoglycan synthesis—the primary constituent of bacterial cell walls. Additionally, the interaction between nisin and lipid II results in the formation of aggregates, leading to pore formation within the cytoplasmic membrane. This, in turn, causes a loss of membrane integrity and functionality.^18^

For *P. aeruginosa*, none of the scaffolds (freeze dried or non-freeze dried) showed any antimicrobial efficacy at any time points (Fig. 10(c and d)). This is due to the difficulty nisin has in penetrating the outer membrane of Gram-negative bacteria and interacting with the precursor lipid II.^18^

### 3.9. Biofilm inhibition assay

**Fig. 11:**
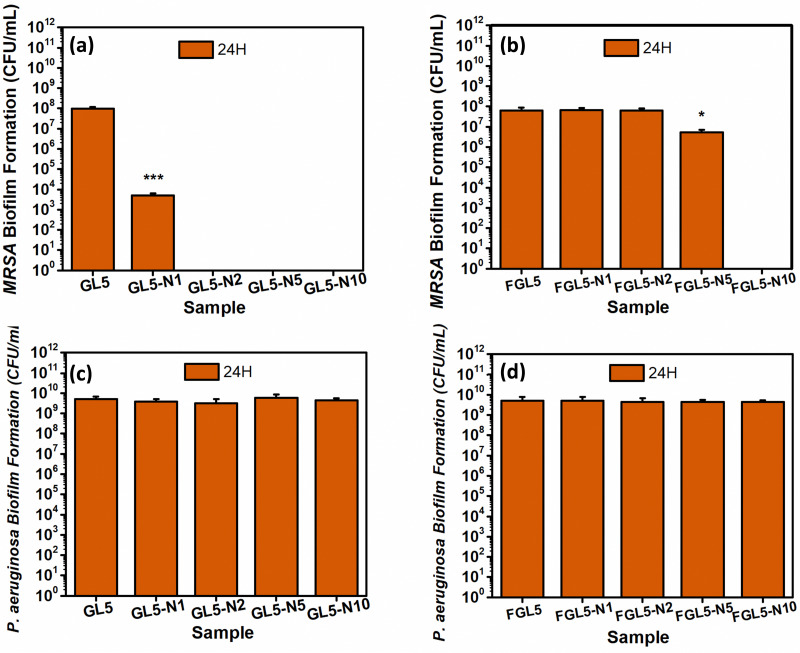
Biofilm inhibition assay of the nisin scaffolds in gel and freeze-dried form at 4- and 24-h time points. (a) Antimicrobial efficacy of the non-freeze-dried scaffolds against *MRSA*. (b) Antimicrobial efficacy of the freeze-dried scaffolds against *MRSA*. (c) Antimicrobial efficacy of the non-freeze-dried scaffolds against *Pseudomonas aeruginosa*. (d) Antimicrobial efficacy of the freeze-dried scaffolds against *Pseudomonas aeruginosa*. *P < 0.05, **P < 0.01, ***P < 0.001 (*N* = *3*, *n* = 3).

In the *MRSA* biofilm assay at the 24-hour time point, it was observed that GL5-N2, GL55-N5, and GL5-N10 could completely kill the bacteria, while GL5-N1 demonstrated a 4-log reduction as shown in Fig. 11(a). For the freeze-dried scaffolds, FGL5-N10 displayed complete bacteria killing, GL5-N5 showed a 1 log reduction, while FGL5-N2 and FGL5-N1 had no antimicrobial efficacy against *MRSA* biofilm formation (Fig. 11(b)). Gelatin, a molecule composed of amino acids, the fundamental units of proteins, serves as a substantial nutritional source for bacteria. Bacteria possess enzymes capable of enzymatically breaking down these amino acids and peptides into simpler molecules that are easily assimilated. This breakdown process provides bacteria with essential resources: firstly, the remaining carbon serves as crucial sources for building new cellular components. Secondly, the nitrogen embedded within the amino acid structure fulfils another critical need by providing bacteria with a source of nitrogen, essential for the construction of vital molecules such as proteins and nucleic acids. Thus, gelatin not only offers bacterial cells a rich substrate for growth but also satisfies their fundamental nutritional requirements through its amino acid composition.^46^

Furthermore, as with the planktonic assay, as expected no antimicrobial activity was observed against *P. aeruginosa* in both scaffold types^47^ (Fig. 11(c and d)).

### 3.10. Stability

**Fig. 12:**
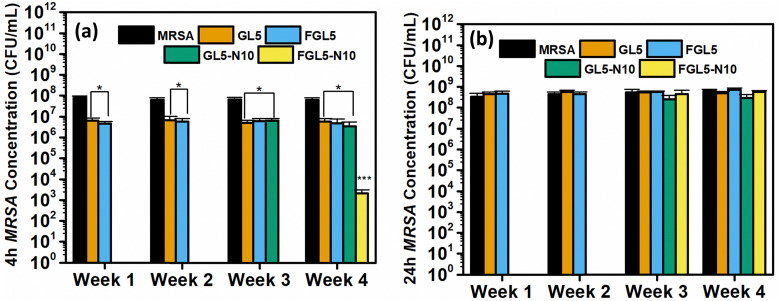
Nisin stability assay of the scaffolds, non-freeze dried and freeze-dried at 4 hour and 24 hours, 4-week duration. (a) Antimicrobial efficacy of the nisin scaffolds at 4 h against *MRSA*. (b) Antimicrobial efficacy of the nisin scaffolds at 24 h against *MRSA*. *P < 0.05, **P < 0.01, ***P < 0.001 (*N* = 3, *n* = 3).

The stability of nisin was evaluated against *MRSA* and *Pseudomonas aeruginosa* at 1, 2, 3, and 4 weeks. The antimicrobial efficacy of GL5, FGL5, GL5-N10 and FGL5-N10 was tested at 4 and 24 hours (Fig. 12).

The stability of nisin was evaluated by examining GL5 and FGL5 scaffolds and the highest concentration of nisin, GL5-N10 and FGL5-N10 and testing their antimicrobial efficacy every week for 4 weeks. As seen in Fig. 12 the freeze-dried and non-freeze-dried scaffolds achieved complete killing of bacteria at both 4 and 24-hour time points in weeks 1 and 2. However, by weeks 3 and 4, the freeze-dried scaffolds showed no antimicrobial efficacy at the 24-hour time point but still demonstrated complete killing at the 4-hour time point in week 3. The non-freeze-dried scaffolds exhibited no antimicrobial efficacy at either time point from week 3 onwards. This suggests that although there is a considerable loss in nisin concentration upon freeze drying, the nisin that is left within the scaffold has a higher stability and longer shelf-life in comparison to the non-freeze-dried counterparts.

## 4. Conclusion

The developed gelatin/nisin hydrogel ink exhibits favourable rheological properties for 3D bioprinting. Comprehensive physicochemical and rheological characterization outlines the impact of scaffold form (non-freeze or freeze-dried) on various parameters, including stability, swelling, degradation, and antimicrobial efficacy. Antimicrobial assays against methicillin-resistant *Staphylococcus aureus* (*MRSA*) and *Pseudomonas aeruginosa* in planktonic form demonstrated the efficacy of the nisin-loaded scaffolds. Distinct responses were observed between the gel and freeze-dried forms, emphasizing the importance of scaffold composition in influencing antimicrobial activity. Moreover, the biofilm inhibition assay showcased the concentration-dependent efficacy against *MRSA* biofilms, reinforcing the potential of these 3D printed scaffolds in preventing bacterial colonization and promoting a sterile environment within tissue-engineered structures. This study provides a foundation for the development of 3D bioprinted scaffolds with inherent antimicrobial properties, showing promise for applications in tissue engineering and regenerative medicine.

## Author contributions

The manuscript was written through contributions from all authors. All authors have given approval to the final version of the manuscript.

## Data availability

Data are available within the article or its ESI.† The data that support the findings are also available on request from the first author (Mateo Dallos Ortega m.dallos@liverpool.ac.uk) and corresponding author (Raechelle D’Sa r.dsa@liverpool.ac.uk).

## Conflicts of interest

The authors declare no competing financial interest.

## Supplementary Material

MA-005-D4MA00544A-s001
