# Supplementary material for: 3D printable gelatin/nisin biomaterial inks for antimicrobial tissue engineering applications
Source: Mater Adv. 2024 Sep 10;5(19):7729–46. doi: 10.1039/d4ma00544a (PMC11385060; doi:10.1039/d4ma00544a)
Supplement: MA-005-D4MA00544A-s001 [file MA-005-D4MA00544A-s001.pdf]

Supplementary Information (SI) for Materials Advances.
This journal is © The Royal Society of Chemistry 2024

3D printable gelatin/nisin biomaterial inks for antimicrobial tissue engineering applications

Mateo Dallos Ortega[1], Jenny Aveyard[1], Alexander Ciupa[2], Robert J Poole[1], David Whetnall[2], Julia G. Behnsen[1]and Raechelle A. D'Sa[1]

[1]School of Engineering, University of Liverpool, Harrison Hughes Building, Brownlow Hill, Liverpool, L69 3GH, UK

[2]Materials Innovation Factory, University of Liverpool, 51 Oxford Street, Liverpool L7 3NY, UK

*Corresponding author: r.dsa@liverpool.ac.uk

Supplementary Information

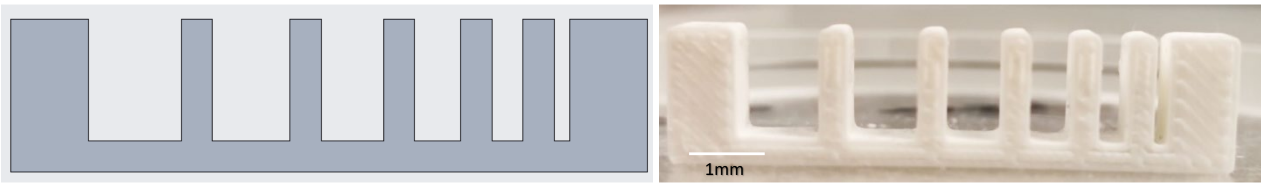

Figure S1: CAD design and printed seven pillar stage.

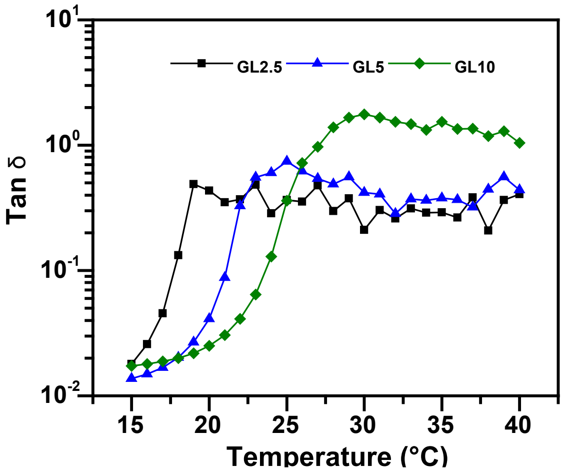

Figure S2: Tan δ values as a function of temperature. Viscoelastic characterization of gelatin inks.

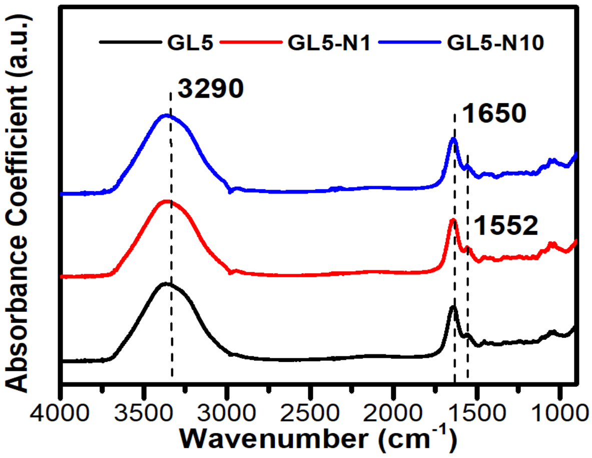

Figure S3: Fourier-transform infrared spectroscopy of GL5, GL5-N1 and GL5-N10 inks.

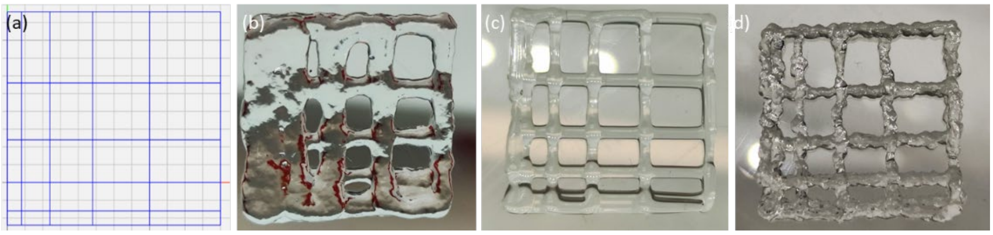

Figure S4: Printed grid patterns to assess diffusion rate percentage. a) 2D design of grid pattern designs. b) GL2.5 ink. c) GL5 ink. D) GL10 ink.

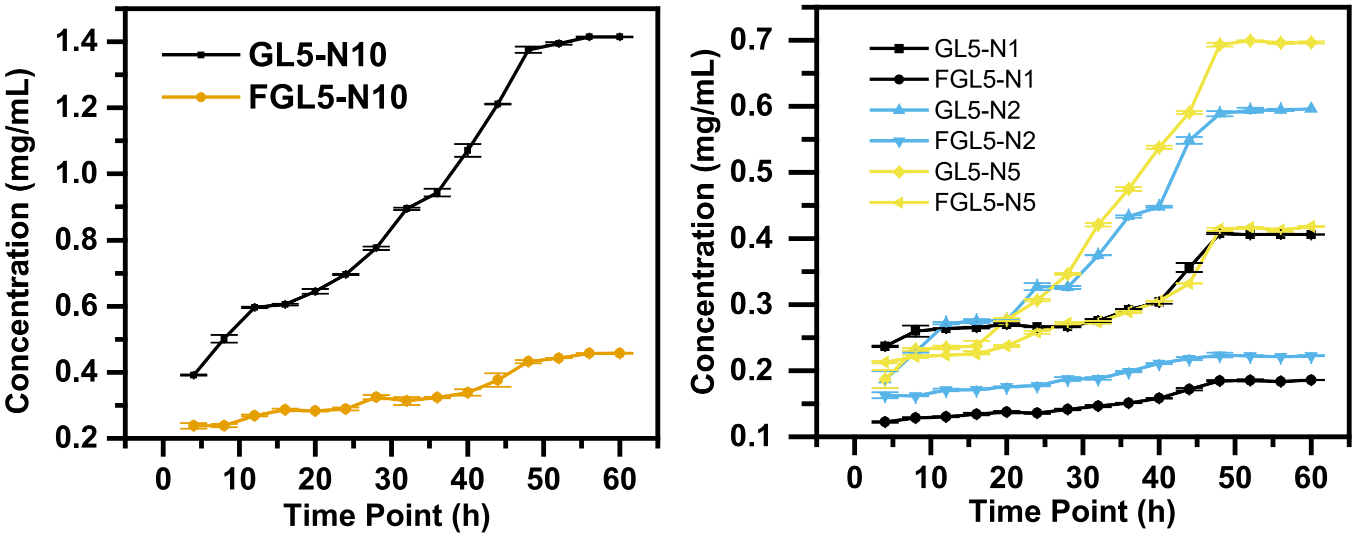

Figure S5: Nisin drug release for all ink concentrations over a 60-hour period measured by high-performance liquid chromatography (HPLC).
